# Using the social amoeba *Dictyostelium* to study the functions of proteins linked to neuronal ceroid lipofuscinosis

**DOI:** 10.1186/s12929-016-0301-0

**Published:** 2016-11-24

**Authors:** Robert J. Huber

**Affiliations:** Department of Biology, Trent University, 2140 East Bank Drive, Peterborough, ON K9J 7B8 Canada

**Keywords:** *Dictyostelium discoideum*, Neuronal ceroid lipofuscinosis, Batten disease, Growth, Development, Calcium, Lysosome, Model organism

## Abstract

Neuronal ceroid lipofuscinosis (NCL), also known as Batten disease, is a debilitating neurological disorder that affects both children and adults. Thirteen genetically distinct genes have been identified that when mutated, result in abnormal lysosomal function and an excessive accumulation of ceroid lipofuscin in neurons, as well as other cell types outside of the central nervous system. The NCL family of proteins is comprised of lysosomal enzymes (PPT1/CLN1, TPP1/CLN2, CTSD/CLN10, CTSF/CLN13), proteins that peripherally associate with membranes (DNAJC5/CLN4, KCTD7/CLN14), a soluble lysosomal protein (CLN5), a protein present in the secretory pathway (PGRN/CLN11), and several proteins that display different subcellular localizations (CLN3, CLN6, MFSD8/CLN7, CLN8, ATP13A2/CLN12). Unfortunately, the precise functions of many of the NCL proteins are still unclear, which has made targeted therapy development challenging. The social amoeba *Dictyostelium discoideum* has emerged as an excellent model system for studying the normal functions of proteins linked to human neurological disorders. Intriguingly, the genome of this eukaryotic soil microbe encodes homologs of 11 of the 13 known genes linked to NCL. The genetic tractability of the organism, combined with its unique life cycle, makes *Dictyostelium* an attractive model system for studying the functions of NCL proteins. Moreover, the ability of human NCL proteins to rescue gene-deficiency phenotypes in *Dictyostelium* suggests that the biological pathways regulating NCL protein function are likely conserved from *Dictyostelium* to human. In this review, I will discuss each of the NCL homologs in *Dictyostelium* in turn and describe how future studies can exploit the advantages of the system by testing new hypotheses that may ultimately lead to effective therapy options for this devastating and currently untreatable neurological disorder.

## Background

### *Dictyostelium* as a model system for studying human neurological disorders

**Fig. 1:**
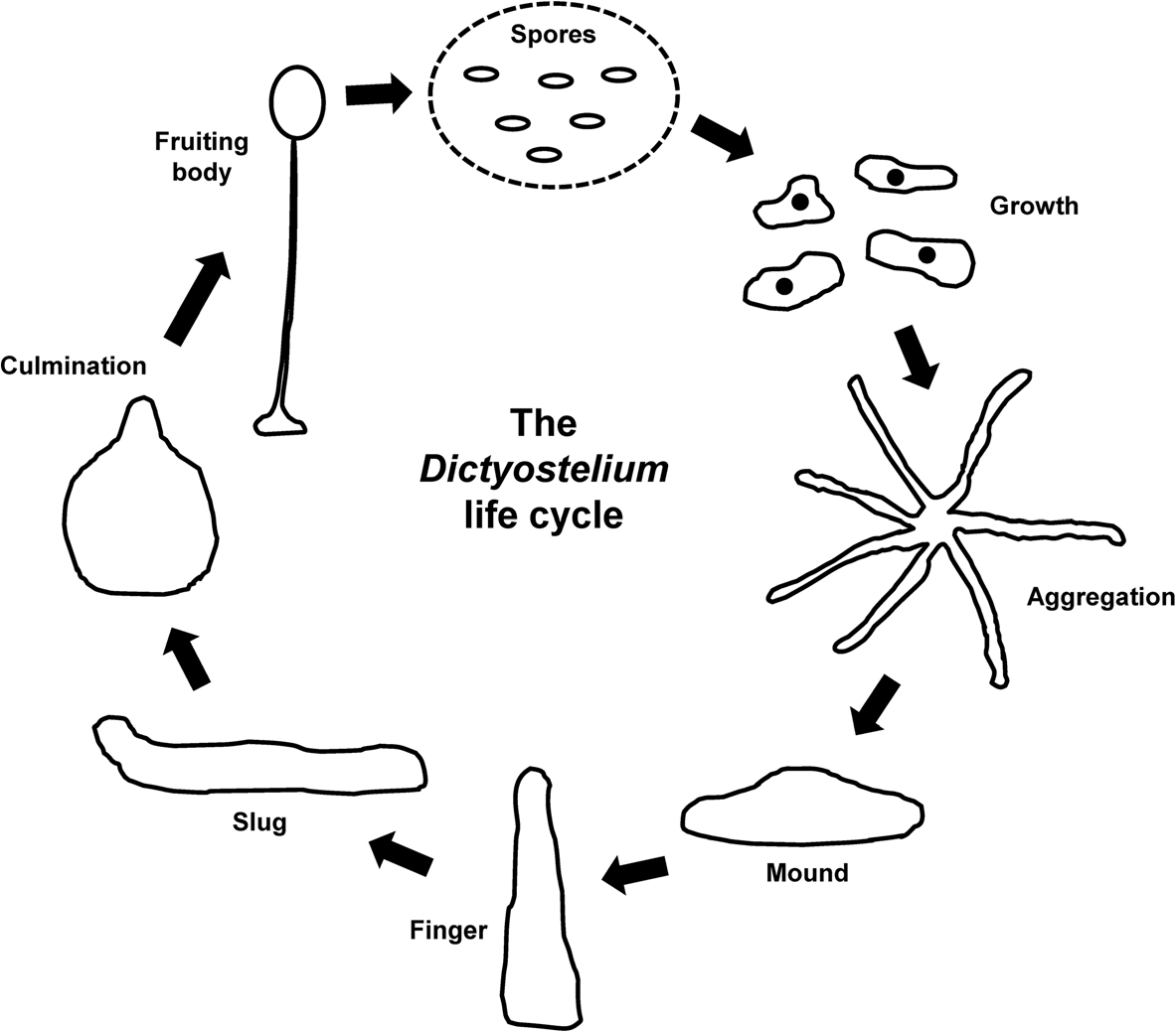
The life cycle of *Dictyostelium*. During growth, single cells feed on bacteria. Upon starvation, cells undergo chemotactic aggregation towards cAMP to form a multicellular mound. The mound then forms a finger, which falls on the surface to generate a motile pseudoplasmodium, or slug. During culmination, terminal differentiation of pre-stalk and pre-spore cells forms a fruiting body composed of a mass of viable spores supported atop a slender stalk. When a food source becomes available, the spores germinate allowing the cells to restart the life cycle

The social amoeba *Dictyostelium discoideum* is a fascinating microbe that has emerged as a valuable model organism for biomedical and human disease research. This model eukaryote, which has historically been used to study basic cell function and multicellular development, undergoes a 24-h asexual life cycle comprised of both single-cell and multicellular phases [1] (Fig. 1). As a result, it is an excellent system for studying a variety of cellular and developmental processes, including lysosome function and intracellular trafficking and signalling [2, 3]. In nature, *Dictyostelium* feeds and grows as single cells (Fig. 1). When prompted by starvation, cells undergo chemotactic aggregation towards cAMP to form a multicellular aggregate (i.e., a mound), which then undergoes a series of morphological changes to form a motile multicellular pseudoplasmodium, also referred to as a slug (Fig. 1). Cells within the slug then terminally differentiate into either stalk or spore to form a fruiting body [4] (Fig. 1). Unlike immortalized mammalian cells that have been removed from their respective tissues, *Dictyostelium* represents a true organism in the cellular state that retains all of its dynamic physiological processes. Moreover, the cellular processes and signalling pathways that regulate the behaviour of *Dictyostelium* cells are remarkably similar to those observed in metazoan cells, indicating that findings from *Dictyostelium* are highly likely to be translatable to more complex eukaryotic systems [5].

*Dictyostelium* is recognized as an excellent model system for studying human neurological disorders, including epilepsy, lissencephaly, Parkinson’s disease, Alzheimer’s disease, and Huntington’s disease [6–10]. *Dictyostelium* also contains the highest content of prion-like proteins of all organisms investigated to date [11, 12]. Intriguingly, prion-like proteins known to generate insoluble deposits in other eukaryotic cells, do not aggregate in *Dictyostelium* [11, 12]. Instead these proteins accumulate in the nucleus and are broken down by the ubiquitin–proteasome system suggesting that *Dictyostelium* has undergone specific adaptations that allow it to efficiently regulate its prion-like proteome [11, 12]. Thus, understanding the pathways involved in preventing protein aggregation in *Dictyostelium* could provide novel routes to tackling neurological disorders characterized by protein aggregation (e.g., Alzheimer’s, Parkinson’s, and Huntington’s disease).

### *Dictyostelium* as a model system for studying neuronal ceroid lipofuscinosis

Recently, *Dictyostelium* has emerged as a significant model system for studying neuronal ceroid lipofuscinosis (NCL) [13–15]. NCL, also known as Batten disease, is a neurological disorder that affects both children and adults [16]. The disease, which is the most common form of neurodegeneration in children, affects all ethnicities worldwide. The highest rates of incidence are observed in Northern European populations and Newfoundland, Canada [17–20]. Over 430 mutations have been documented in thirteen genetically distinct NCL genes, which are categorized based on the age of onset and pathological features [16, 21, 22] (Table 1). However, more genes likely remained to be identified as some patients who present with NCL-like symptoms do not carry mutations in any of the known NCL genes. Clinical manifestations of the disease include the progressive loss of vision, mental ability, and motor function, as well as epileptic seizures and a reduced lifespan [23]. At the cellular level, NCL disorders characteristically display aberrant lysosomal function and an excessive accumulation of ceroid lipofuscin in neurons and other cell types, with pathology often seen outside of the central nervous system [22].

**Table 1:** List of genes linked to NCL in humans

Protein	Disease
PPT1/CLN1	Infantile NCL
TPP1/CLN2	Late-infantile NCL
CLN3	Juvenile NCL
DNAJC5/CLN4	Adult-onset NCL, Kufs disease, Parry disease
CLN5	Late-infantile NCL (Finnish variant)
CLN6	Variant late-infantile NCL
MFSD8/CLN7	Late-infantile NCL (Turkish variant)
CLN8	Northern epilepsy (epilespy, progressive with mental retardation, EPMR)
CTSD/CLN10	Congenital, neonatal and late infantile NCL
PGRN/CLN11	Adult-onset NCL, frontotemporal dementia in heterozygotes
ATP13A2/CLN12	Juvenile-onset NCL
CTSF/CLN13	Adult-onset NCL
KCTD7/CLN14	Infantile NCL

Substantial molecular advances have been made in the past decade, which has made a significant impact on genetic diagnosis and our understanding of the biology underlying the NCL disorders. The NCL family of proteins is comprised of lysosomal enzymes (PPT1/CLN1, TPP1/CLN2, CTSD/CLN10, CTSF/CLN13), proteins that peripherally associate with membranes (DNAJC5/CLN4, KCTD7/CLN14), a soluble lysosomal protein (CLN5), a protein that is present in the secretory pathway (PGRN/CLN11), and several transmembrane proteins that display different subcellular localizations (CLN3, CLN6, MFSD8/CLN7, CLN8, ATP13A2/CLN12) [16, 21]. Unfortunately, the precise functions of many of the NCL proteins are still unknown [21]. As a result, new innovative approaches are critically needed to identify the primary functions of these proteins, which will hopefully lead to targeted therapy development for this devastating and currently untreatable disease. Interestingly, recent genetic advances now clearly support a strong overlap of NCL phenotypes with later onset neurodegenerative diseases, including Parkinson’s and frontotemporal dementia [24, 25]. Therefore, studying the cellular mechanisms underlying NCL protein function may provide fresh new insight into these and other related neurological disorders.

**Fig. 2:**
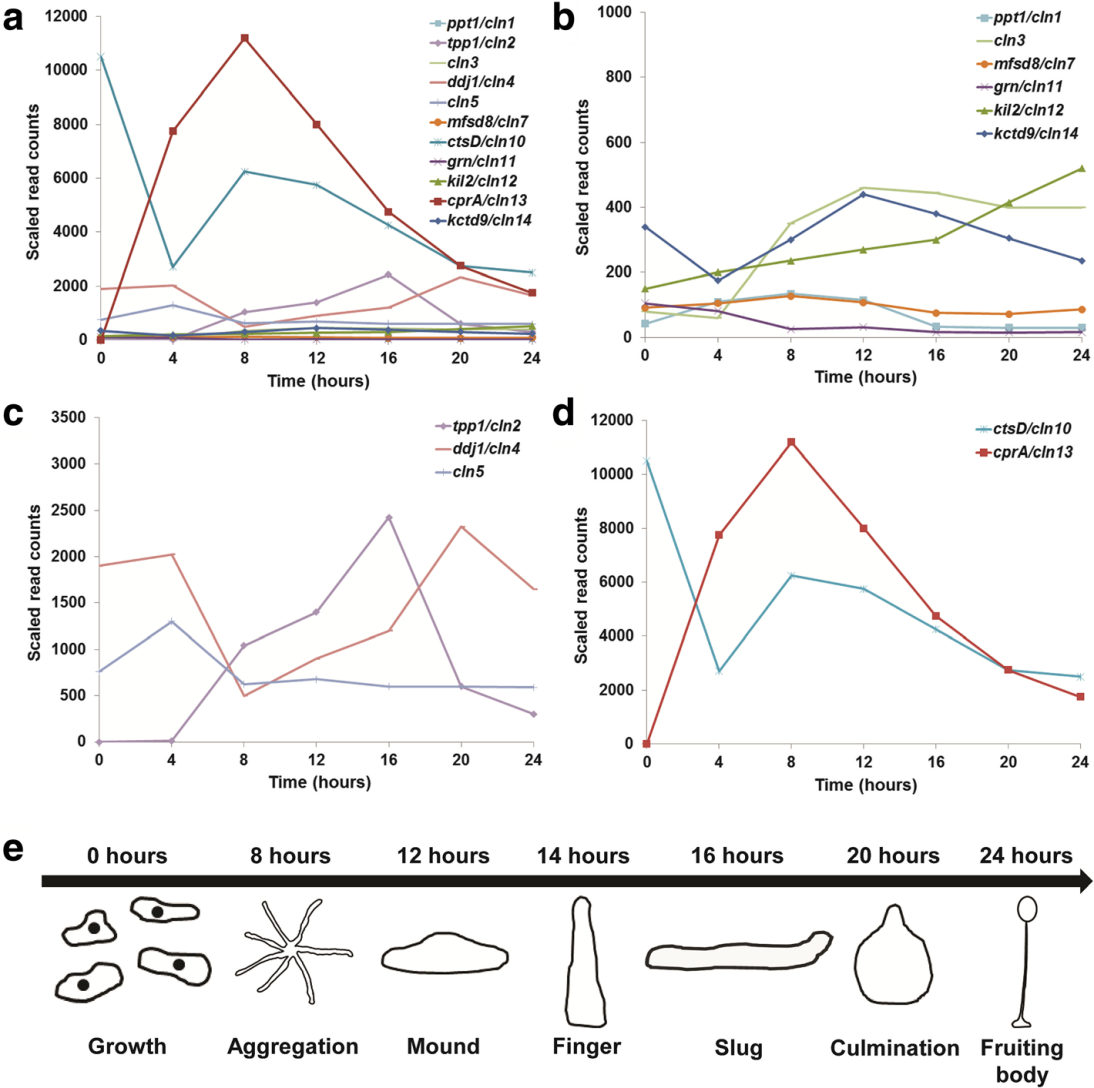
Gene expression analysis of NCL homologs in *Dictyostelium*. RNA-Seq data was obtained from dictyExpress (http://www.dictyexpress.biolab.si) [31] and re-plotted using Microsoft Excel. Genes with similar magnitudes of scaled read counts were grouped together on the same plot. **a** Expression profiles of all NCL homologs. **b** Expression profiles of *ppt1*, *cln3*, *mfsd8*, *grn*, *kil2*, *kctd9*. **c** Expression profiles of *tpp1*, *ddj1*, and *cln5*. **d** Expression profiles of *ctsD* and *cprA*. **e** Timeline of the various stages of *Dictyostelium* development. Images are not drawn to scale

Homologs of NCL proteins have been identified and studied in several model organisms and this work has significantly enhanced our understanding of the localization and functions of NCL proteins in humans [26–28]. The *Dictyostelium* genome contains homologs of 11 of the 13 known NCL genes, but does not contain homologs of *CLN6* or *CLN8* (Table 2). Of all the NCL proteins, these are the only ER-resident membrane proteins [29, 30]. By comparison, *Dictyostelium* contains more homologs of NCL proteins than other model organisms including budding and fission yeast, the nematode *Caenorhabditis elegans*, and the fruit fly *Drosophila melanogaster*, but fewer than zebrafish (*Danio rerio*) [26–28] (Table 3). While the functions of some of the homologs in *Dictyostelium* have been investigated using gene-deficiency models, knockout mutants for several of the genes have yet to be generated (e.g., *ppt1/cln1*, *ddj1/cln4*, *cln5*, *mfsd8/cln7*, *grn/cln11*, *cprA/cln13*). Moreover, of the genes that have been studied, many have not been investigated in relation to NCL (e.g., *ctsD/cln10*, *kil2/cln12*, *kctd9/cln14*). The mRNA expression profiles of the *Dictyostelium* NCL homologs suggest different roles during the life cycle, with some being required for growth, and others for development [31] (Fig. 2). Moreover, many of the NCL homologs were detected in a proteomic profile of the macropinocytic pathway suggesting that the proteins share common functions or participate in the same biological pathway or process [32]. In this review, I will discuss each of the NCL homologs in *Dictyostelium* in turn, and highlight how we can use this model organism to provide fresh new insight into NCL protein function. While the focus of this review will be on the *Dictyostelium* homologs of human TPP1/CLN2, CLN3, CTSD/CLN10, ATP13A2/CLN12, CTSF/CLN13, and KCTD7/CLN14, the potential to use *Dictyostelium* to elucidate the functions of the other NCL proteins will also be discussed.

**Table 2:** *Dictyostelium* proteins homologous to human NCL proteins

Human protein	Size (aa)	*Dictyostelium* homolog (dictyBase ID)	Size (aa)	Region of similarity (aa)	Identities (%)	Positives (%)	*E*-value
PPT1/CLN1 Accession: NP_000301	306	Ppt1 (DDB0233890)	303	273	46	69	8.00E-80
TPP1/CLN2 Accession: NP_000382	563	Tpp1 (DDB0234303)	600	552	36	52	4.00E-91
CLN3 Accession: NP_000077	438	Cln3 (DDB0233983)	421	429	27	45	3.00E-31
DNAJC5/CLN4 Accession: NP_079495	198	Ddj1 (DDB0215016)	411	69	57	76	2.00E-18
CLN5 Accession: NP_006484	407	Cln5 (DDB0234077)	322	301	30	47	2.00E-27
CLN6 Accession: NP_060352	311	No homolog					
MFSD8/CLN7 Accession: NP_689991	518	Mfsd8 (DDB0307149)	498	530	29	47	1.00E-41
CLN8 Accession: NP_061764	286	No homolog					
CTSD/CLN10 Accession: NP_001900	412	CtsD (DDB0215012)	383	359	47	66	9.00E-91
PGRN/CLN11 Accession: NP_002078	593	Grn (DDB0238428)	130	47	55	68	2.00E-08
ATP13A2/CLN12 Accession: NP_071372	1180	Kil2 (DDB0237611)	1158	1089	35	53	1.00E-175
CTSF/CLN13 Accession: NP_003784	484	CprA (DDB0201647)	343	340	37	55	6.00E-64
KCTD7/CLN14 Accession: NP_694578	289	Kctd9 (DDB0231824)	488	96	38	58	2.00E-11

The amino acid sequences of human NCL proteins were inputted into the BLASTp server of dictyBase (http://www.dictybase.org). The following parameters were set: *E*-value, 1000; Matrix, BLOSUM62; Filter, no

**Table 3:** Established and putative homologs of NCL proteins in other model organisms

Human Protein	*S. cerevisiae* (budding yeast) *S. pombe* (fission yeast)	*C. elegans* (nematode)	*D. melanogaster* (fruit fly)	*D. rerio* (zebrafish)
PPT1/CLN1 Accession: NP_000301	Yes (*S. pombe*) [26–28]	Yes [26]	Yes [26–28]	Yes [27, 28]
TPP1/CLN2 Accession: NP_000382	No	No	No	Yes [27, 28]
CLN3 Accession: NP_000077	Yes [26–28]	Yes [26–28]	Yes [26–28]	Yes [27, 28]
DNAJC5/CLN4 Accession: NP_079495	Yes [a]	Yes [28]	Yes [c]	Yes [27, 28]
CLN5 Accession: NP_006484	No	No	No	Yes [27, 28]
CLN6 Accession: NP_060352	No	No	No	Yes [27, 28]
MFSD8/CLN7 Accession: NP_689991	No	Yes [b]	Yes [27, 28]	Yes [27, 28]
CLN8 Accession: NP_061764	No	No	No	Yes [27, 28]
CTSD/CLN10 Accession: NP_001900	Yes [26–28]	Yes [26, 28]	Yes [26–28]	Yes [27, 28]
PGRN/CLN11 Accession: NP_002078	No	Yes [b]	No	Yes [27, 28]
ATP13A2/CLN12 Accession: NP_071372	Yes [28],[a]	Yes [b]	Yes [c]	Yes [27, 28]
CTSF/CLN13 Accession: NP_003784	No	Yes [b]	Yes [c]	Yes [27, 28]
KCTD7/CLN14 Accession: NP_694578	No	Yes [b]	Yes [c]	Yes [27, 28]

The information contained within this table is described in detail elsewhere [26–28]. The presence and absence of homologs in each organism was confirmed with NCBI BLASTp [a], WormBase BLASTp [b], and FlyBase BLASTp [c]

### Ppt1, the *Dictyostelium* homolog of human PPT1/CLN1

Mutations in palmitoyl-protein thioesterase 1 (*PPT1/CLN1)* cause one of the two infantile forms of NCL and the most severe subtype of the disease [21, 23] (Table 1). Clinical symptoms present between the ages of 1 and 2, and usually results in death between the ages of 8 and 11 [33]. However late infantile, juvenile, and adult forms of this NCL subtype have also been reported [34–38]. The *PPT1* gene is conserved from yeast to human [26–28] (Table 3). PPT1 localizes to the lysosomal matrix, cytoplasmic vesicles, the endoplasmic reticulum (ER), lipid rafts, and presynaptic areas in neurons [21] (Table 4). The targets of PPT1-mediated de-palmitoylation and the precise function of the protein are still not known, however the protein is thought to be involved in apoptosis, cholesterol metabolism, and the recycling of synaptic vesicles [21] (Table 5).

**Table 4:** Localization of NCL homologs in *Dictyostelium* and other systems

*Dictyostelium* homolog	dictyBase ID	Localization in *Dictyostelium*	Localization of human protein (or homologs in other systems)
Ppt1	DDB0233890	Extracellular space [40] Macropinocytic pathway [32]	Cytoplasmic vesicles [21] Endoplasmic reticulum [21] Lipid rafts [21] Lysosomal matrix [21] Presynaptic areas in neurons [21]
Tpp1	DDB0234303	Lysosome [14]	Endoplasmic reticulum [21] Lysosomal matrix [21]
Cln3	DDB0233983	CV system [13, 15] Endocytic pathway [13, 15] Macropinocytic pathway [32]	Late endosomal membrane [21] Lysosomal membrane [21]
Ddj1	DDB0215016	Centrosome [69] Macropinocytic pathway [32] Phagosome [70]	Cell membrane [68] Cytoplasm [21] Melanosomes [67] Synaptic vesicles in neurons [21] Secretory granules in endocrine, neurocrine and exocrine cells [21] Vesicular membranes [21]
Cln5	DDB0234077	Macropinocytic pathway [32]	Extracellular space [76] Lysosomal matrix [21, 75]
Mfsd8	DDB0307149	Macropinocytic pathway [32]	Lysosomal membrane [21]
CtsD	DDB0215012	Endocytic pathway (e.g., phagosome, lysosome) [81–83] Macropinocytic pathway [32]	Extracellular space [21, 79, 80] Lysosomal matrix [21]
Grn	DDB0238428	Not known	Extracellular space [21, 97]
Kil2	DDB0237611	Macropinocytic pathway [32] Phagosomal membrane [102]	Lysosomal membrane [21, 99, 100] Multi-vesicular bodies [21, 99, 100]
CprA	DDB0201647	Not known	Lysosomal matrix [21]
Kctd9	DDB0231824	Not known	Cell membrane [21, 114] Punctate cytoplasmic distributions [21, 114]

**Table 5:** Cellular processes modulated by NCL homologs in *Dictyostelium* and other systems

*Dictyostelium* homolog	dictyBase ID	Processes modulated in *Dictyostelium*	Knockout mutant generated	Processes modulated by human protein (or homologs in other systems)
Ppt1	DDB0233890	Phagocytosis [41]	No	Apoptosis [21] Cholesterol metabolism [21] Phagocytosis [42] Recycling of synaptic vesicles [21]
Tpp1	DDB0234303	Autophagy [14] Pre-spore cell differentiation [14] Timing of mid-stage development [14]	Yes [14]	Endocytosis [21] Macroautophagy [21] TNF-α-induced apoptosis [21]
Cln3	DDB0233983	Cell proliferation [13] Pinocytosis [13] Protein secretion and cleavage [13] Cell adhesion [15] Timing of mid-stage development [15] Timing of late-stage development [15] Response to bacterial pathogens [66]	Yes [13]	Apoptosis [21] Autophagy [21] Calcium homeostasis [21] Cell cycle control [21] Endocytosis [21] Intracellular trafficking [21] Lysosomal pH homeostasis [21] Osmoregulation [21]
Ddj1	DDB0215016	Phagocytosis [70]	No	Presynaptic endocytosis and exocytosis [21]
Cln5	DDB0234077	Not known	No	Apoptosis [21] Cell growth [21] Myelination [21] Sorting and recycling of lysosomal receptors [21] Sphingolipid transport and synthesis [21]
Mfsd8	DDB0307149	Not known	No	Predicted to have a role in: Transport of small substrates across the lysosomal membrane [21]
CtsD	DDB0215012	Bacterial killing [86] Cell death [88, 89] Timing of early-stage development [81]	Yes [79]	Apoptosis [21, 90] Autophagy [21, 91] Cell migration [85] Innate immune response [87]
Grn	DDB0238428	Not known	No	Autophagy [21, 97] Cell migration [21, 97] Embryogenesis [21, 97] Inflammation [21, 97] Tumorigenesis [21, 97]
Kil2	DDB0237611	Bacterial killing [102, 105] Predation on yeast [104]	Yes [100]	Predicted to have a role in: Lysosomal degradation [21] pH and biometal homeostasis within lysosomes [21] Transport across cellular membranes [21]
CprA	DDB0201647	Early-stage development [108] Osmoregulation [110]	No	Autophagy [21] Cell immunity [21, 113] Lipoprotein degradation [21] Proteasome degradation [21]
Kctd9	DDB0231824	Not known	Yes [118]	Predicted to have a role in: Hyperpolarization of the neuronal cell membrane [21] Proteasome degradation [21]

The *Dictyostelium* homolog of human *PPT1*, *ppt1*, is composed of three exons that encode a 303 amino acid, 34 kDa protein (Ppt1; DDB0233890) (Table 2). Like PPT1, the *Dictyostelium* homolog contains a signal peptide for secretion suggesting that it may function extracellularly (SignalP 4.0) [39]. Not surprisingly, the enzyme has been detected in the macropinocytic pathway of *Dictyostelium* as well as in conditioned media from developing cells [32, 40] (Table 4). *ppt1* expression increases during the early stages of development and reaches its peak level after 8 h [31] (Fig. 2b). Expression then decreases dramatically during mid-stage development and remains low during terminal differentiation and fruiting body formation (Fig. 2b). These results suggest that Ppt1 may be important for processes that occur during the early stages of development, specifically cAMP chemotaxis and aggregation. A knockout mutant has not yet been generated to study the function of Ppt1 in *Dictyostelium*. However, a previous study that analyzed genome-wide transcriptional changes during bacterial feeding and growth suggests that Ppt1 may function during phagocytosis, which supports data from mice linking PPT1 function to phagocyte infiltration following neuronal cell death [41, 42] (Table 5).

### Tpp1, the *Dictyostelium* homolog of human TPP1/CLN2

Mutations in tri-peptidyl peptidase 1 (*TPP1/CLN2)* cause a late infantile form of NCL [21, 23] (Table 1). Clinical symptoms present between the ages of 2 and 4, with death typically occurring between the ages of 6 and 15 [43]. In addition to NCL, mutations in *TPP1* have also been linked to autosomal recessive spinocerebellar ataxia 7 (SCAR7) [44]. The TPP1 protein localizes to the lysosomal matrix and the ER, functions as a serine protease, and has been linked to endocytosis, macroautophagy, and TNF-α-induced apoptosis [21] (Tables 4 and 5). While TPP1 is highly conserved in vertebrates, homologs are not present in classical model organisms such as yeast, the nematode, and the fruit fly [45] (Table 3). As a result, the NCL research community has been unable to exploit the relative advantages of these systems to study TPP1 function. Fortunately, the *Dictyostelium* genome encodes a homolog of TPP1 which was recently characterized [14]. The *Dictyostelium* homolog, *tpp1*, is composed of one exon that encodes a 600 amino acid, 67 kDa protein (Tpp1; DDB0234303) (Table 2). Tpp1 contains peptidase S8 and S53 domains as well as a putative signal peptide for secretion (SignalP 4.0) [39]. However the protein has not been detected in conditioned media from developing *Dictyostelium* cells [40]. *tpp1* expression increases during the early-to-mid stages of development reaching peak levels after 16 h [31] (Fig. 2c). Expression then decreases dramatically during the later stages of development (Fig. 2c). The function of the protein, which is enriched in pre-spore cells, was recently characterized [14, 46]. Importantly, *Dictyostelium* Tpp1 shares many attributes with human TPP1. As in human cells, *Dictyostelium* Tpp1 localizes to the lysosome [14, 22] (Table 4). Auto-fluorescent bodies are observed in *Dictyostelium tpp1* ^*−*^ cells during mid-stage development, which is similar to the auto-fluorescent material observed in human cells expressing mutated TPP1 [14, 22]. However the composition of the aggregated material in *Dictyostelium* cells remains to be identified. *tpp1* ^*−*^ cells develop precociously and show a reduced ability to cleave target substrates and form spores [14] (Table 5). Similar to phenotypes observed in mammalian cell models, Tpp1-deficient cells also show defects in autophagy [14] (Table 5). More specifically, in response to starvation, *tpp1* ^*−*^ cells display reduced cell size and viability when compared to wild-type [14]. In support of its localization to the lysosome, Tpp1-deficienct cells display strongly impaired development when treated with the lysosome-perturbing drug chloroquine [14]. Importantly, Phillips and Gomer [14] showed that this phenotype can be suppressed by a secondary mutation in *stpA* (suppressor of Tpp1, DDB_G0282973), which encodes a protein that shares some sequence similarity to mammalian oxysterol-binding proteins. This finding indicates that *Dictyostelium* has the potential to make substantial contributions to NCL protein research, specifically by exploiting the genetic tractability of the organism to identify other secondary mutations that suppress NCL phenotypes.

### Cln3, the *Dictyostelium* homolog of human CLN3

The most common subtype of NCL is caused by loss-of-function mutations in ceroid lipofuscinosis neuronal 3 (*CLN3)*, which is one of the two juvenile forms of the disease [21, 23] (Table 1). Clinical symptoms present between the ages of 5 and 10 and result in premature death usually in the late teenage years or early 20s [22, 33]. The human *CLN3* gene encodes a 438 amino acid multi-pass transmembrane protein that is predicted to contain 6 transmembrane domains [47]. The gene is conserved from yeast to human and has been studied using a number of systems and cell models [26–28] (Table 3). The CLN3 protein, which localizes to late endosomes and the lysosomal membrane, has been linked to autophagy, apoptosis, intracellular trafficking, lysosomal pH homeostasis, cell cycle control, osmoregulation, and calcium homeostasis [21] (Tables 4 and 5). However, the precise function of CLN3 and the mechanism by which the protein affects these processes remains unknown [26–28].

The *Dictyostelium* homolog, *cln3*, is composed of three exons that encode a 421 amino acid, 47 kDa protein (Cln3; DDB0233983) predicted to contain 10–11 transmembrane domains (Table 2). Importantly, amino acid residues that are post-translationally modified in human CLN3 are conserved in the *Dictyostelium* homolog, as are residues mutated in patients with the disease [13]. *cln3* expression increases dramatically during the early stages of development reaching peak levels after 12 h [31] (Fig. 2b). Expression then remains high during the mid-to-late stages of development (Fig. 2b). Loss of Cln3 results in aberrant secretion and cleavage of autocrine proliferation repressor A (AprA) and counting factor-associated protein A (CfaD) during growth [13]. As a consequence, Cln3-deficient cells display an increased rate of proliferation compared to wild-type [13] (Table 5). During the early stages of development, the aggregation of *cln3* ^*−*^ cells is delayed and aberrant, but this phenotype is not due to reduced chemotaxis, alterations in the expression or localization of the cAMP receptor (CarA), or aberrant expression of genes linked to cAMP signal transduction [15] (Table 5). However, *cln3* ^*−*^ cells do display reduced cell-substrate and cell-cell adhesion, which correlates with a decreased intracellular amount of the cell adhesion protein contact site A (CsaA), and an increased amount of soluble calcium-dependent cell adhesion molecule (CadA) in conditioned media [15] (Table 5). These results suggest that the reduced adhesion of *cln3* ^*−*^ cells to the substrate and to each other causes the delayed and aberrant aggregation. As proof-of-concept, aberrant adhesion has also been observed in mammalian cells lacking functional CLN3 (e.g., mouse cerebellar cells, human neuronal progenitor cells derived from induced pluripotent stem cells generated from patient fibroblasts) (Huber, Atwal, Chandrachud, Cotman, unpublished observations). Finally, during the mid-to-late stages of development, *cln3* ^*−*^ cells develop precociously and *cln3* ^*−*^ slugs display enhanced migration [13] (Table 5).

In *Dictyostelium*, Cln3 localizes to both the contractile vacuole (CV) system and the endocytic pathway (late endosomes/lysosomes), and was detected in a proteomic profile of the macropinocytic pathway [13, 15, 32] (Table 4). Like other protists, the *Dictyostelium* CV system functions primarily as an osmoregulatory organelle [48]. However roles in ion homeostasis, vesicular trafficking, and unconventional protein secretion have also been reported [48–50]. Importantly, defects in osmoregulation have been reported in mammalian cell models lacking functional CLN3 [51–53]. In *Dictyostelium*, the CV system is a major intracellular store of ions, particularly calcium, and is required for cAMP-mediated calcium influx during chemotaxis [48, 54–56]. Calcium has also been shown to function as a chemoattractant during the early stages of *Dictyostelium* development [57]. Moreover, the primary sensor of intracellular calcium, calmodulin, localizes predominantly on the CV system [50, 58]. Previous work has shown that Cln3-deficiency phenotypes during development can be rescued by chelating calcium with EGTA [13, 15]. More specifically, calcium chelation rescues the aberrant streaming and aggregation of *cln3* ^*−*^ cells, the precocious formation and enhanced migration of *cln3* ^*−*^ slugs, and the precocious formation of *cln3* ^*−*^ fruiting bodies [13, 15]. These findings suggest that *cln3* ^*−*^ cells may inappropriately buffer calcium from the environment, and are consistent with studies in mammalian systems that have reported altered ion homeostasis, most notably calcium, in the absence of functional CLN3 [59–64]. Given its localization to the CV system, *Dictyostelium* presents an excellent system for studying the possible function of Cln3 in calcium homeostasis. As discussed above, Cln3-deficiency alters the extracellular levels of the cell proliferation regulators AprA and CfaD during growth, and CadA during the early stages of development [13, 15]. Intriguingly, CadA is secreted via an unconventional pathway involving the CV system [65]. Coupled with the localization of GFP-Cln3 to the CV system, these observations suggest that Cln3 may function to regulate unconventional protein secretion via the CV system during *Dictyostelium* growth and development. Together these results indicate that further study of Cln3 localization to the CV system could provide valuable new insight into the primary function of CLN3 in human cells.

In *Dictyostelium*, Cln3-deficiency causes pleiotropic effects that can be rescued through the expression of either *Dictyostelium* Cln3 or human CLN3, thus confirming that the function of CLN3 is evolutionarily conserved [13, 15]. Moreover, the localization of Cln3 to the CV system in *Dictyostelium* suggests that this model organism could provide an excellent system for assessing the role of the protein in osmotic stress, vesicular trafficking, ion homeostasis, and unconventional protein secretion. On a final note, *Dictyostelium* has been used as a model system to study the virulence of *Pseudomonas aeruginosa*, which is one of the most relevant human opportunistic bacterial pathogens. *Dictyostelium* cells infected with one of two strains of *P. aeruginosa* (PAO1 and PA14) cause a decrease in the expression of *cln3* suggesting that Cln3 is also involved in the response of *Dictyostelium* cells to bacterial pathogens [66] (Table 5).

### Ddj1, the *Dictyostelium* homolog of DNAJC5/CLN4

Mutations in *DNAJC5/CLN4* cause an adult-onset form of NCL that is also known as Kufs disease or Parry disease [21, 23] (Table 1). *DNAJC5* is conserved from yeast to human and encodes cysteine-string protein alpha (CSPα), which is a protein chaperone that localizes to the cytoplasm and associates with vesicular membranes, synaptic vesicles in neurons, and secretory granules in endocrine, neurocrine, and exocrine cells [21, 27, 28] (Tables 3 and 4). It has also been detected at the cell membrane and in melanosomes [67, 68] (Table 4). The disease-causing mutations in CSPα occur within the highly conserved cysteine-string region that is responsible for membrane binding and oligomerization [21].

The *Dictyostelium* homolog of DNAJC5, Ddj1 (DnaJ homolog 1, DDB0215016), localizes to both the cytoplasm and cell cortex, and was detected in proteomic profiles of the centrosome and macropinocytic pathway [32, 69] (Tables 2 and 4). The *ddj1* gene is composed of two exons that encode a 411 amino acid, 46 kDa protein. *ddj1* expression is highest during growth, the first 4 h of development, and fruiting body formation [31] (Fig. 2c). Expression decreases substantially during aggregation reaching its lowest level after 8 h of development, after which time the expression begins to increase (Fig. 2c). The peak expression level occurs after 20 h of development (Fig. 2c). Together, these observations suggest that the protein likely functions during growth and terminal differentiation. A *Dictyostelium ddj1* knockout mutant has not yet been generated, however the function of the protein has been linked to phagocytosis in *Dictyostelium* [70] (Table 5).

### Cln5, the *Dictyostelium* homolog of human CLN5

Mutations in ceroid lipofuscinosis neuronal 5 (*CLN5*) cause a late-infantile form of NCL [21, 23]. However, early juvenile and adult cases have also been reported [71–73] (Table 1). While this subtype had previously been referred to as the Finnish variant, it now appears in other regions of world [74]. The CLN5 protein localizes to the lysosomal matrix, but has also been detected in conditioned media from cultured cells [21, 75, 76] (Table 4). While the precise function of CLN5 in human cells is not known, it is proposed to function in sphingolipid transport and synthesis, myelination, cell growth, apoptosis, and the sorting and recycling of lysosomal receptors [21] (Table 5). Like CLN2, homologs of CLN5 do not exist in yeast, the nematode, or the fruit fly (Table 3).

The *Dictyostelium* homolog of human *CLN5*, *cln5*, is composed of three exons that encode a 322 amino acid, 37 kDa protein (Cln5; DDB0234077) (Table 2). While the localization of Cln5 in *Dictyostelium* has not yet been revealed, the protein was detected in a proteomic analysis of the macropinocytic pathway [32] (Table 4). Interestingly, like human CLN5, the *Dictyostelium* homolog contains a signal peptide for secretion (SignalP 4.0) [39] and has been detected in conditioned media from developing cells (Huber, manuscript in preparation). *cln5* expression levels indicate that the protein may play an important role during the early stages of development [31] (Fig. 2c). Expression increases dramatically during the first 4 h of development, decreases significantly during the next four hours, and then remains low during the remaining stages of development (Fig. 2c). Future work to generate and characterize a *cln5* knockout mutant should reveal Cln5-dependent processes in *Dictyostelium* that may provide insight into the precise function of the protein in human cells. This is especially important considering that classical model organisms such as yeast, the nematode, and the fruit fly lack homologs of CLN5 (Table 3).

### Mfsd8, the *Dictyostelium* homolog of human MFSD8/CLN7

Mutations in major facilitator superfamily domain-containing protein 8 (*MFSD8*), also known as *CLN7*, cause a late-infantile form of NCL that was previously referred to as the Turkish variant [21, 23] (Table 1). The protein, which contains 12 transmembrane domains, localizes to the lysosomal membrane and is predicted to play a role in the transport of small substrates across the lysosomal membrane [21] (Tables 4 and 5). However, the precise function of the protein and its substrates are still not known. Homologs of MFSD8 are present in the nematode, the fruit fly, and zebrafish, but absent in yeast [27, 28] (Table 3).

The *Dictyostelium* homolog of human *MFSD8*, *mfsd8*, is composed of one exon that encodes a 498 amino acid, 55 kDa protein (Mfsd8; DDB0307149) (Table 2). Proteomic analyses have localized the protein to the macropinocytic pathway [32] (Table 4). Although a knockout mutant has not yet been generated in *Dictyostelium*, RNA-seq studies have provided some insight into when during the life cycle the protein may function. *mfsd8* expression increases during the first 8 h of development [31] (Fig. 2b) suggesting that the protein functions during cAMP chemotaxis and aggregation. Expression then decreases between 8 and 20 h before rising slightly during fruiting body formation (Fig. 2b). By generating and characterizing a *Dictyostelium mfsd8* knockout mutant, future research may be able to provide insight into the primary function of MFSD8 that is lost in NCL patients.

### CtsD, the *Dictyostelium* homolog of human CTSD/CLN10

Mutations in cathepsin D (*CTSD*), also known as *CLN10*, have been linked to aging and neurodegeneration, including congenital, neonatal, and late infantile forms of NCL [21, 23, 77] (Table 1). Juvenile forms of this NCL subtype have also been reported [78]. CTSD is an aspartic protease that is conserved from yeast to human [26–28] (Table 3). It localizes to the lysosomal matrix and is linked to aberrant apoptosis and autophagy [21] (Tables 4 and 5). The enzyme, which contains a peptidase A1 domain and a signal peptide for secretion, has also been detected extracellularly, where its function is linked to a number of human cancers (SignalP 4.0) [39, 79] (Table 4). Proteomic profiling has also detected extracellular CTSD in aortic samples, where it was found to be loosely bound to the matrix [80] (Table 4).

The *Dictyostelium* homolog of human *CTSD*, *ctsD*, is composed of two exons that encode a 383 amino acid, 41 kDa protein (CtsD; DDB0215012) that is present throughout the endocytic pathway, including maturing phagosomes and lysosomes [32, 81–83] (Tables 2 and 4). Like human CTSD, the *Dictyostelium* homolog contains a signal peptide for secretion (SignalP 4.0) [39] and has been detected in conditioned media from developing cells (Huber, manuscript in preparation). The mRNA expression profile of *ctsD* indicates that the protein functions primarily during growth, where its expression in the highest [31] (Fig. 2d). *ctsD* expression decreases dramatically during the first four hours of development before rising slightly between 4 and 8 h (Fig. 2d). Expression then steadily decreases during the remaining stages of development (Fig. 2d). *ctsD* ^*−*^ cells display no overt phenotypes during growth or development, except for a slight delay in aggregation [81] (Table 5). Interestingly, *ctsD* expression is downregulated 10-fold in *srfB* ^*−*^ cells [84]. SrfB (serum response factor B) is a DNA-binding protein that similar to *ctsD*, is expressed in growth-phase cells as well as during the later stages of aggregation [84]. *srfB* ^*−*^ cells display decreased proliferation, aberrant cytokinesis, increased pinocytosis, abolished streaming, and precocious aggregation [84]. The defects in streaming and aggregation can be rescued by manually stimulating cells with pulses of cAMP suggesting that cAMP signaling is aberrant in *srfB* ^*−*^ cells [84]. These phenotypes, coupled with the similar expression profiles of *srfB* and *ctsD*, the dramatic reduction in the expression of *ctsD* in *srfB* ^*−*^ cells, and the delayed aggregation of *ctsD* ^*−*^ cells suggest that CtsD is required for optimal migration and aggregation in *Dictyostelium*. This is supported by studies in mammalian cells that have reported reduced migration of CTSD-deficient cells due to aberrant organization of cytoskeletal components, which if present in neurons, could have negative effects on neurogenesis, maintenance of neuronal polarity and shape, and migration [85] (Table 5).

In addition to studies on SrfB function, other work in *Dictyostelium* has also provided insight into CtsD function, specifically its role in bacterial killing and cell death. During infection with *Salmonella typhimurium*, the agent of food-borne gastroenteritis, *Dictyostelium* cells activate a defense response involving the upregulation of a number of cysteine proteases and cathepsins, including CtsD [86] (Table 5). Interestingly, studies in mice have linked CTSD function to the innate immune response against *Listeria monocytogenes* [87]. In *Dictyostelium*, research suggests that CtsD is involved in staurosporine-induced cell death and functions with calpains to facilitate cell dismantling during oxidative stress-induced cell death [88, 89] (Table 5). Intriguingly, CTSD is linked to staurosporine-induced apoptosis in human fibroblasts and activates autophagy to inhibit oxidative stress-induced cell death in human cancer cells [90, 91]. Staurosporine induces apoptosis in a number of mammalian cell lines, activating both caspase-dependent and caspase-independent types of cell death [92]. Caspase-independent cell death occurs through the release of lysosomal enzymes into the cytosol [93]. This is significant since the *Dictyostelium* genome does not encode caspases and instead undergoes caspase-independent cell death, which results in the release of lysosomal enzymes such as CtsD into the cytoplasm [94, 95]. Together, these results have provided insight into the functions of CtsD during the *Dictyostelium* life cycle. Future research using this model organism may be able to assess the effects of NCL-causing *CTSD* mutations on CtsD-dependent processes in *Dictyostelium*.

### Grn, the *Dictyostelium* homolog of human PGRN/CLN11

Mutations in *PGRN* (progranulin), also referred to as *CLN11*, cause an adult-onset form of NCL in humans [21, 23] (Table 1). Interestingly, heterozygote mutations in *PGRN* also cause frontotemporal dementia [96]. PGRN is a multi-domain protein that is secreted and proteolytically cleaved to generate 6-kDa cleavage products called granulins (GRNs) (1–7) [21, 97] (Table 4). The primary functions of GRNs are still not known, however they are proposed to function in autophagy, embryogenesis, cell motility, inflammation, and tumorigenesis [21, 97] (Table 5). Homologs of GRNs are present in the nematode and zebrafish, but absent in yeast and the fruit fly [27, 28] (Table 3).

The *Dictyostelium* homolog of human GRN, Grn, is highly similar in sequence to human GRNs 1–7 (Table 2). The *grn* gene is composed of two exons that encode a 130 amino acid, 14 kDa protein (Grn; DDB0238428). The mRNA expression profile of *grn* indicates that the protein likely functions during growth where its expression is the highest [31] (Fig. 2b). Expression then decreases dramatically during development reaching its lowest level between 16 and 24 h suggesting that it may not be required for processes that occur during the mid-to-late stages of *Dictyostelium* development (e.g., terminal differentiation) (Fig. 2b). Although a *grn* knockout mutant has not yet been generated, future work aimed at creating a *Dictyostelium* Grn-deficiency model may be able to provide insight into the primary function of GRNs, and how mutations in *PGRN* cause NCL and frontotemporal dementia in humans.

### Kil2, the *Dictyostelium* homolog of human ATP13A2/CLN12

Another juvenile-onset form of NCL is caused by mutations in *ATP13A2*, which is also known as *CLN12* [21, 23] (Table 1). ATP13A2 contains 10 transmembrane domains and belongs to a family of P-type ATPases that are involved in the active transport of cations, heavy metals, and lipids across cellular membranes [98]. ATP13A2 is conserved from yeast to human and localizes to the lysosomal membrane and multi-vesicular bodies [21, 27, 28, 99, 100] (Tables 3 and 4). The precise function of the protein is not yet known however it is predicted to transport heavy metals, cations, and lipids across cellular membranes, and to be involved in degrading material and maintaining pH and biometal homeostasis within lysosomes [21] (Table 5). Interestingly, ATP13A2 also protects against α-synuclein toxicity in several models of Parkinson’s disease [101].

The *Dictyostelium* homolog of ATP13A2 is Kil2 (DDB0237611), which is a V-type P-ATPase that contains 10 transmembrane domains [102] (Table 2). The *kil2* gene is composed of two exons that encode an 1158 amino acid, 131 kDa protein that localizes to the phagosomal membrane and is present in the macropinocytic pathway [32, 102] (Table 4). The mRNA expression profile of *kil2* suggests that the protein functions primarily during development, specifically during terminal differentiation. Expression increases steadily during development reaching peak levels during fruiting body formation [31] (Fig. 2b).

*Dictyostelium* serves as an excellent model system for studying the cellular mechanisms involved in the degradation of ingested bacteria and yeast [103, 104], and previous work investigating this process has provided some insight into the function of Kil2. In humans, the efficient ingestion and killing of bacteria by phagocytic cells is necessary to protect the human body from infectious microorganisms. Kil2-deficient cells show reduced phagosomal activity compared to wild-type cells and are not able to kill ingested *Klebsiella pneumoniae* bacteria [102, 105] (Table 5). However these defects can be restored by supplementing the medium with magnesium suggesting that Kil2 functions as a magnesium pump to maintain the optimal concentration of magnesium in phagosomes, and to ensure the activity of phagosomal proteases [102]. Intriguingly, *kil2* ^*−*^ cells are still able to kill several other species of bacteria (e.g., *B. subtilis* and *P. aeruginosa*) suggesting that the response of cells to ingested bacteria likely involves a diversity of proteins with different specificities [102]. More recently, Kil2 function has also been linked to the predation of *Dictyostelium* amoeba on yeast [104]. Since Kil2 appears to function as a magnesium pump in *Dictyostelium*, and ATP13A2 is proposed to function in maintaining biometal homeostasis in human lysosomes, these results support the use of *Dictyostelium* as a model system for studying the function of this protein. Thus, future work in *Dictyostelium* may be able to provide new insight into the function of ATP13A2 in humans and how mutations in this gene cause NCL.

### CprA, the *Dictyostelium* homolog of human CTSF/CLN13

Mutations in cathepsin F *(CTSF),* also known as *CLN13*, have been linked to another adult-onset form of NCL [21, 23] (Table 1). CTSF is a cysteine protease that belongs to the peptidase C1 family of proteases. The protein localizes to the lysosomal matrix, and has been proposed to function in autophagy, proteasome degradation, cell immunity, and lipoprotein degradation [21] (Tables 4 and 5). CTSF contains a signal peptide for secretion and has been detected extracellularly (SignalP 4.0) [39, 106] (Table 4). Homologs of CTSF are present in the nematode, the fruit fly, and zebrafish, but absent in yeast [27, 28] (Table 3).

CprA (cysteine proteinase A; DDB0201647) is the *Dictyostelium* homolog of human CTSF (Table 2). The gene, which shares a high degree of homology to plant and animal sulphydryl proteinases, is composed of six exons that encode a 343 amino acid, 39 kDa protein [107]. *cprA* is not expressed during the growth phase of the life cycle [31]. However, its expression, which is inducible by extracellular cAMP, increases dramatically during the early stages of development [31, 108] (Fig. 2d). By 10–12 h, *cprA* mRNA comprises approximately 1% of the total cellular mRNA [109]. While the expression of *cprA* steadily decreases during the remaining stages of development, *cprA* has been identified as a pre-stalk specific gene [31, 46, 109] (Fig. 2d). It has been suggested that CprA functions to digest proteins during development to provide amino acids and a source of energy for developing cells [109]. A *cprA* knockout mutant has not yet been generated however previous studies have provided some insight into the function of the protein. *cprA* expression was shown to be induced during hyperosmotic stress with 200 mM sorbitol suggesting that protein functions during the osmotic stress response [110] (Table 5). *cprA* expression is also upregulated in *rpkA* ^*−*^ cells during starvation [111]. RpkA (receptor phosphatidylinositol kinase A) is seven-transmembrane G-protein coupled receptor (GPCR) that contains a C-terminal phosphatidylinositol-4-phosphate 5-kinase (PIP5K) domain (http://www.dictybase.org). Importantly, RpkA localizes to late phagosomes and is involved in the phagocytosis of infectious bacteria [112], which adheres to the proposed function of CTSF in cell immunity in mammalian cells [113] (Table 5). At low density, *rpkA* ^*−*^ cells fail to aggregate due to their inability to respond to cAMP and conditioned media factor (CMF) [111]. Given the increased expression of *cprA* in *rpkA* ^*−*^ cells, and the dramatic increase in expression of *cprA* during the early stages of development, these results suggest that CprA is required for cAMP chemotaxis and aggregation in *Dictyostelium*. Finally, like human CTSF, CprA contains a signal peptide for secretion and has been detected in conditioned media from developing cells (SignalP 4.0) [39] (Huber, manuscript in preparation). Together, these results have provided insight into the function of CprA during the *Dictyostelium* life cycle. Further examination of its localization in *Dictyostelium*, and the creation of a CprA-deficiency model, may be able to provide additional insight into the role of CTSF in human disease.

### Kctd9, the *Dictyostelium* homolog of human KCTD7/CLN14

Mutations in potassium channel tetramerization-domain 7 (*KCTD7*), also known as *CLN14*, have been linked to another infantile form of NCL [21, 23, 114] (Table 1). In humans, the KCTD family of proteins consists of 26 members with mostly unknown functions [115]. Mutations in *KCTD7* cause vision loss, progressive myoclonic epilepsy, progressive decline in cognition and motor skills, and a reduced lifespan [114]. NCL-type storage material is also observed in patient samples [114]. In a wild-type mouse cerebellar cell line, KCTD7 fused to GFP localizes to the cell membrane, as well as punctate distributions within the cytoplasm [114] (Table 4). The precise function of the protein is not yet known however it is predicted to be involved in proteasome degradation and hyperpolarization of the neuronal cell membrane [21] (Table 5). Homologs of KCTD7 are present in the nematode, the fruit fly, and zebrafish, but absent in yeast [27, 28] (Table 3).

Kctd9 (DDB0231824) is the *Dictyostelium* homolog of human KCTD7 (Table 2). The protein contains four penta-peptide repeats, a double-courtin domain, and is highly similar to vertebrate potassium channel tetramerization domain-containing proteins. The gene is composed of one exon that encodes a 488 amino acid, 53 kDa protein. The mRNA expression profile of *kctd9* suggests that the protein functions during the mid-stages of development. *kctd9* expression decreases during the early stages of development reaching its lowest level after 4 h [31] (Fig. 2b). Expression then increases significantly to reach its peak level after 12 h, and is followed by a steady decrease in expression between 12 and 24 h of development (Fig. 2b). Although the effect of Kctd9-deficiency during growth and development has not yet been studied, gene expression studies have provided some insight into its function during sexual development. In addition to the asexual life cycle, *Dictyostelium* can also undergo sexual reproduction to form macrocysts [116]. During this alternative life cycle, phermonal interactions generate fusion-competent cells, which then undergo cell and pronuclear fusion through a process involving cAMP chemotaxis. *kctd9* ^*−*^ cells show no obvious phenotypes during macrocyst formation, however *kctd9* expression is enriched in gametes (e.g., sexually mature, fusion-competent cells) [117, 118] suggesting that the protein may be involved in membrane fusion during sexual development. The research community would benefit from future work examining the precise function of Kctd9 during membrane fusion as well as examining the effect of Kctd9 loss on processes that occur during the *Dictyostelium* asexual life cycle.

## Conclusions

Accumulated evidence indicates that *Dictyostelium* could provide unprecedented opportunities to elucidate the normal functions of NCL proteins and the signal transduction regulating their activities. This is especially true for those proteins that have no clear homologs in other classical model organisms. Many of the NCL homologs in *Dictyostelium* were detected in a proteomic profile of the macropinocytic pathway, which supports the suggestion that NCL proteins share common functions or participate in the same biological pathway or process [32, 119] (Table 4). While the functions of some of the NCL homologs in *Dictyostelium* have been previously studied, many have not been investigated in relation to human disease. A major benefit of using *Dictyostelium* to study NCL protein function is the ability to knockout multiple genes in a single cell line using homologous recombination [120]. Importantly, this allows researchers to study epistatic relationships between genes and will allow future studies to assess potential functional redundancies between the NCL homologs in *Dictyostelium*. However, one must acknowledge that using *Dictyostelium* as model system for studying NCL protein function does present some limitations. For instance, while *Dictyostelium* does develop into a true multicellular organism, it contains a limited number of cell types that may present challenges when attempting to relate gene-deficiency phenotypes in *Dictyostelium* to pathologies in specific tissues or organs in humans. Since *Dictyostelium* lacks a central nervous system, discoveries made in *Dictyostelium* must be evaluated in the relevant mammalian cell type. Nonetheless, the ability of human NCL proteins to rescue deficiency phenotypes in *Dictyostelium* [13–15] suggests that the biological pathways regulating the functions of NCL proteins are likely conserved from *Dictyostelium* to human. In addition to normal function models, future studies can use *Dictyostelium* to assess the effects of newly discovered mutations on NCL protein function, which will greatly improve our understanding of the molecular basis of the NCL disorders, and will hopefully aid in the development of therapies that rescue the molecular defects in NCL patients.

## Abbreviations

AprA: Autocrine proliferation repressor A
cAMP: cyclic AMP
CfaD: Counting factor-associated protein A
CLN: Ceroid lipofuscinosis neuronal
CMF: Conditioned media factor
CprA: Cysteine proteinase A
CSPα: Cysteine-string protein alpha
CTSD: Cathepsin D
CTSF: Cathepsin F
CV: Contractile vacuole
Ddj1: DnaJ homolog 1
ER: Endoplasmic reticulum
GPCR: G protein-coupled receptor
KCTD: Potassium channel tetramerization-domain
MFSD8: Major facilitator superfamily domain-containing protein 8
NCL: Neuronal ceroid lipofuscinosis
PGRN: Progranulin
PIP5K: Phosphatidylinositol-4-phosphate 5-kinase
PPT1: Palmitoyl-protein thioesterase 1
RpkA: Receptor phosphatidylinositol kinase A
SCAR7: Spinocerebellar ataxia 7
SrfB: Serum response factor B
StpA: Suppressor of Tpp1 A
TPP1: Tri-peptidyl peptidase 1

## References

[1] Fey P, Kowal AS, Gaudet P, Pilcher KE, Chisholm RL (2007). Protocols for growth and development of *Dictyostelium discoideum*. Nat Protoc.

[2] Simon M, Plattner H (2014). Unicellular eukaryotes as models in cell and molecular biology: critical appraisal of their past and future value. Int Rev Cell Mol Biol.

[3] Maniak M (2011). *Dictyostelium* as a model for human lysosomal and trafficking diseases. Semin Cell Dev Biol.

[4] Du Q, Kawabe Y, Schilde C, Chen ZH, Schaap P (2015). The evolution of aggregative multicellularity and cell-cell communication in the *Dictyostelia*. J Mol Biol.

[5] Müller-Taubenberger A, Kortholt A, Eichinger L (2013). Simple system—substantial share: the use of *Dictyostelium* in cell biology and molecular medicine. Eur J Cell Biol.

[6] Walker MC, Williams RS (2013). The search for better epilepsy treatments: from slime mould to coconuts. Biochem Soc Trans.

[7] Meyer I, Kuhnert O, Gräf R (2011). Functional analyses of lissencephaly-related proteins in *Dictyostelium*. Semin Cell Dev Biol.

[8] Kortholt A, Gilsbach B, van Haastert, PJM. *Dictyostelium discoideum*: A Model System to Study LRRK2-Mediated Parkinson Disease. In: Dushanova J, editor. Mechanisms in Parkinson’s Disease–Models and Treatments. Croatia: InTech; 2012. p. 293–310.

[9] McMains VC, Myre M, Kreppel L, Kimmel AR (2010). *Dictyostelium* possesses highly diverged presenilin/gamma-secretase that regulates growth and cell-fate specification and can accurately process human APP: a system for functional studies of the presenilin/gamma-secretase complex. Dis Model Mech.

[10] Myre MA, Lumsden AL, Thompson MN, Wasco W, MacDonald ME, Gusella JF (2011). Deficiency of huntingtin has pleiotropic effects in the social amoeba *Dictyostelium discoideum*. PLoS Genet.

[11] Santarriaga S, Petersen A, Ndukwe K, Brandt A, Gerges N, Bruns Scaglione J, Scaglione KM (2015). The social amoeba *Dictyostelium discoideum* is highly resistant to polyglutamine aggregation. J Biol Chem.

[12] Malinovska L, Palm S, Gibson K, Verbavatz JM, Alberti S (2015). *Dictyostelium discoideum* has a highly Q/N-rich proteome and shows an unusual resilience to protein aggregation. Proc Natl Acad Sci U S A.

[13] Huber RJ, Myre MA, Cotman SL (2014). Loss of Cln3 function in the social amoeba *Dictyostelium discoideum* causes pleiotropic effects that are rescued by human CLN3. PLoS One.

[14] Phillips JE, Gomer RH (2015). Partial genetic suppression of a loss-of-function mutant of the neuronal ceroid lipofuscinosis-associated protease TPP1 in *Dictyostelium discoideum*. Dis Model Mech.

[15] Huber RJ, Myre MA, Cotman SL. Aberrant adhesion impacts early development in a *Dictyostelium* model for juvenile neuronal ceroid lipofuscinosis. Cell Adh Migr. in press. 10.1080/19336918.2016.1236179 PMC5569969 27669405

[16] Mole SE, Cotman SL (2015). Genetics of the neuronal ceroid lipofuscinoses (Batten disease). Biochim Biophys Acta.

[17] Andermann E, Jacob JC, Andermann F, Carpenter S, Wolfe L, Berkovic S (1988). The Newfoundland aggregate of neuronal ceroid-lipofuscinosis. Am J Med Genet.

[18] Ju W, Zhong R, Moore S, Moroziewicz D, Currie JR, Parfrey P, Brown WT, Zhong N (2002). Identification of novel CLN2 mutations shows Canadian specific NCL2 alleles. J Med Genet.

[19] Moore SJ, Buckley DJ, MacMillan A, Marshall HD, Steele L, Ray PN, Nawaz Z, Baskin B, Frecker M, Carr SM, Ives E, Parfrey PS (2008). The clinical and genetic epidemiology of neuronal ceroid lipofuscinosis in Newfoundland. Clin Genet.

[20] Williams RE, Mole SE, Williams RE, Goebel HH (2011). Appendix 1: NCL incidence and prevalence data. The Neuronal Ceroid Lipofuscinoses (Batten Disease).

[21] Cárcel-Trullols J, Kovács AD, Pearce DA (2015). Cell biology of the NCL proteins: What they do and don’t do. Biochim Biophys Acta.

[22] Radke J, Stenzel W, Goebel HH (2015). Human NCL Neuropathology. Biochim Biophys Acta.

[23] Schulz A, Kohlschütter A, Mink J, Simonati A, Williams R (2013). NCL diseases–clinical perspectives. Biochim Biophys Acta.

[24] Deng H, Xiu X, Jankovic J (2015). Genetic convergence of Parkinson’s disease and lysosomal storage disorders. Mol Neurobiol.

[25] Dearborn JT, Harmon SK, Fowler SC, O’Malley KL, Taylor GT, Sands MS, Wozniak DF (2015). Comprehensive functional characterization of murine infantile Batten disease including Parkinson-like behavior and dopaminergic markers. Sci Rep.

[26] Phillips SN, Muzaffar N, Codlin S, Korey CA, Taschner PE, de Voer G, Mole SE, Pearce DA (2006). Characterizing pathogenic processes in Batten disease: use of small eukaryotic model systems. Biochim Biophys Acta.

[27] Bond M, Holthaus SM, Tammen I, Tear G, Russell C (2013). Use of model organisms for the study of neuronal ceroid lipofuscinosis. Biochim Biophys Acta.

[28] Faller KM, Gutierrez-Quintana R, Mohammed A, Rahim AA, Tuxworth RI, Wager K, Bond M (2015). The neuronal ceroid lipofuscinoses: Opportunities from model systems. Biochim Biophys Acta.

[29] Heine C, Koch B, Storch S, Kohlschütter A, Palmer DN, Braulke T (2004). Defective endoplasmic reticulum-resident membrane protein CLN6 affects lysosomal degradation of endocytosed arylsulfatase A. J Biol Chem.

[30] Lonka L, Kyttälä A, Ranta S, Jalanko A, Lehesjoki AE (2000). The neuronal ceroid lipofuscinosis CLN8 membrane protein is a resident of the endoplasmic reticulum. Hum Mol Genet.

[31] Rot G, Parikh A, Curk T, Kuspa A, Shaulsky G, Zupan B (2009). dictyExpress: a *Dictyostelium* discoideum gene expression database with an explorative data analysis web-based interface. BMC Bioinformatics.

[32] Journet A, Klein G, Brugière S, Vandenbrouck Y, Chapel A, Kieffer S, Bruley C, Masselon C, Aubry L (2012). Investigating the macropinocytic proteome of *Dictyostelium* amoebae by high-resolution mass spectrometry. Proteomics.

[33] Haltia M, Goebel HH (2013). The neuronal ceroid-lipofuscinoses: a historical introduction. Biochim Biophys Acta.

[34] Bonsignore M, Tessa A, Di Rosa G, Piemonte F, Dionisi-Vici C, Simonati A, Calamoneri F, Tortorella G, Santorelli FM (2006). Novel CLN1 mutation in two Italian sibs with late infantile neuronal ceroid lipofuscinosis. Eur J Paediatr Neurol.

[35] Mitchison HM, Hofmann SL, Becerra CH, Munroe PB, Lake BD, Crow YJ, Stephenson JB, Williams RE, Hofman IL, Taschner PE, Martin JJ, Philippart M, Andermann E, Andermann F, Mole SE, Gardiner RM, O’Rawe AM (1998). Mutations in the palmitoyl-protein thioesterase gene (PPT; CLN1) causing juvenile neuronal ceroid lipofuscinosis with granular osmiophilic deposits. Hum Mol Genet.

[36] Khan A, Chieng KS, Baheerathan A, Hussain N, Gosalakkal J (2013). Novel CLN1 mutation with atypical juvenile neuronal ceroid lipofuscinosis. J Pediatr Neurosci.

[37] Kalviainen R, Eriksson K, Losekoot M, Sorri I, Harvima I, Santavuori P, Jarvela I, Autti T, Vanninen R, Salmenpera T, van Diggelen OP (2007). Juvenile-onset neuronal ceroid lipofuscinosis with infantile CLN1 mutation and palmitoyl-protein thioesterase deficiency. Eur J Neurol.

[38] Ramadan H, Al-Din AS, Ismail A, Balen F, Varma A, Twomey A, Watts R, Jackson M, Anderson G, Green E, Mole SE (2007). Adult neuronal ceroid lipofuscinosis caused by deficiency in palmitoyl protein thioesterase 1. Neurology.

[39] Petersen TN, Brunak S, von Heijne G, Nielsen H (2011). SignalP 4.0: discriminating signal peptides from transmembrane regions. Nat Methods.

[40] Bakthavatsalam D, Gomer RH (2010). The secreted proteome profile of developing *Dictyostelium discoideum* cells. Proteomics.

[41] Sillo A, Bloomfield G, Balest A, Balbo A, Pergolizzi B, Peracino B, Skelton J, Ivens A, Bozzaro S (2008). Genome-wide transcriptional changes induced by phagocytosis or growth on bacteria in *Dictyostelium*. BMC Genomics.

[42] Zhang Z, Lee YC, Kim SJ, Choi MS, Tsai PC, Saha A, Wei H, Xu Y, Xiao YJ, Zhang P, Heffer A, Mukherjee AB (2007). Production of lysophosphatidylcholine by cPLA2 in the brain of mice lacking PPT1 is a signal for phagocyte infiltration. Hum Mol Genet.

[43] Santavuori P (1988). Neuronal ceroid-lipofuscinoses in childhood. Brain Dev.

[44] Sun Y, Almomani R, Breedveld GJ, Santen GWE, Aten E, Lefeber DJ, Hoff JI, Brusse E, Verheijen FW, Verdijk RM, Kriek M, Oostra B, Breuning MH, Losekoot M, den Dunnen JT, van de Warrenburg BP, Maat-Kievit AJA (2013). Autosomal recessive spinocerebellar ataxia 7 (SCAR7) is caused by variants in TPP1, the gene involved in classic late-infantile neuronal ceroid lipofuscinosis 2 disease (CLN2 disease). Hum Mutat.

[45] Wlodawer A, Li M, Gustchina A, Oyama H, Dunn BM, Oda K (2003). Structural and enzymatic properties of the sedolisin family of serine-carboxyl peptidases. Acta Biochim Pol.

[46] Iranfar N, Fuller D, Sasik R, Hwa T, Laub M, Loomis WF (2001). Expression patterns of cell-type-specific genes in *Dictyostelium*. Mol Biol Cell.

[47] The International Batten Disease Consortium (1995). Isolation of a novel gene underlying Batten disease, CLN3. Cell.

[48] Plattner H (2013). Contractile vacuole complex—its expanding protein inventory. Int Rev Cell Mol Biol.

[49] Kinseth MA, Anjard C, Fuller D, Guizzunti G, Loomis WF, Malhotra V (2007). The Golgi-associated protein GRASP is required for unconventional protein secretion during development. Cell.

[50] Sriskanthadevan S, Brar SK, Manoharan K, Siu CH (2013). Ca^(2+)^ -calmodulin interacts with DdCAD-1 and promotes DdCAD-1 transport by contractile vacuoles in *Dictyostelium* cells. FEBS J.

[51] Stein CS, Yancey PH, Martins I, Sigmund RD, Stokes JB, Davidson BL (2010). Osmoregulation of ceroid neuronal lipofuscinosis type 3 in the renal medulla. Am J Physiol Cell Physiol.

[52] Getty A, Kovács AD, Lengyel-Nelson T, Cardillo A, Hof C, Chan CH, Pearce DA (2013). Osmotic stress changes the expression and subcellular localization of the Batten disease protein CLN3. PLoS One.

[53] Tecedor L, Stein CS, Schultz ML, Farwanah H, Sandhoff K, Davidson BL (2013). CLN3 loss disturbs membrane microdomain properties and protein transport in brain endothelial cells. J Neurosci.

[54] Moniakis J, Coukell MB, Janiec A (1999). Involvement of the Ca2 +-ATPase PAT1 and the contractile vacuole in calcium regulation in *Dictyostelium discoideum*. J Cell Sci.

[55] Malchow D, Lusche DF, Schlatterer C, De Lozanne A, Müller-Taubenberger A (2006). The contractile vacuole in Ca^2+^-regulation in *Dictyostelium*: its essential function for cAMP-induced Ca^2+^-influx. BMC Dev Biol.

[56] Bozzaro S, Buracco S, Peracino B (2013). Iron metabolism and resistance to infection by invasive bacteria in the social amoeba *Dictyostelium discoideum*. Front Cell Infect Microbiol.

[57] Scherer A, Kuhl S, Wessels D, Lusche DF, Raisley B, Soll DR (2010). Ca^2+^ chemotaxis in *Dictyostelium discoideum*. J Cell Sci.

[58] Zhu Q, Clarke M (1992). Association of calmodulin and an unconventional myosin with the contractile vacuole complex of *Dictyostelium discoideum*. J Cell Biol.

[59] Boriack RL, Bennett MJ (2001). CLN-3 protein is expressed in the pancreatic somatostatin-secreting delta cells. Eur J Paediatr Neurol.

[60] An Haack K, Narayan SB, Li H, Warnock A, Tan L, Bennett MJ (2011). Screening for calcium channel modulators in CLN3 siRNA knock down SH-SY5Y neuroblastoma cells reveals a significant decrease of intracellular calcium levels by selected L-type calcium channel blockers. Biochim Biophys Acta.

[61] Warnock A, Tan L, Li C, An Haack K, Narayan SB, Bennett MJ (2013). Amlodipine prevents apoptotic cell death by correction of elevated intracellular calcium in a primary neuronal model of Batten disease (CLN3 disease). Biochem Biophys Res Commun.

[62] Chandrachud U, Walker MW, Simas AM, Heetveld S, Petcherski A, Klein M, Oh H, Wolf P, Zhao WN, Norton S, Haggarty SJ, Lloyd-Evans E, Cotman SL (2015). Unbiased cell-based screening in a neuronal cell model of Batten disease highlights an interaction between Ca^2+^ homeostasis, autophagy, and CLN3 protein function. J Biol Chem.

[63] Chang JW, Choi H, Kim HJ, Jo DG, Jeon YJ, Noh JY, Park WJ, Jung YK (2007). Neuronal vulnerability of CLN3 deletion to calcium-induced cytotoxicity is mediated by calsenilin. Hum Mol Genet.

[64] Grubman A, Pollari E, Duncan C, Caragounis A, Blom T, Volitakis I, Wong A, Cooper J, Crouch PJ, Koistinaho J, Jalanko A, White AR, Kanninen KM (2014). Deregulation of biometal homeostasis: the missing link for neuronal ceroid lipofuscinoses?. Metallomics.

[65] Sesaki H, Wong EF, Siu CH (1997). The cell adhesion molecule DdCAD-1 in *Dictyostelium* is targeted to the cell surface by a nonclassical transport pathway involving contractile vacuoles. J Cell Biol.

[66] Carilla-Latorre S, Calvo-Garrido J, Bloomfield G, Skelton J, Kay RR, Ivens A, Martinez JL, Escalante R (2008). *Dictyostelium* transcriptional responses to Pseudomonas aeruginosa: common and specific effects from PAO1 and PA14 strains. BMC Microbiol.

[67] Chi A, Valencia JC, Hu Z-Z, Watabe H, Yamaguchi H, Mangini NJ, Huang H, Canfield VA, Cheng KC, Yang F, Abe R, Yamagishi S, Shabanowitz J, Hearing VJ, Wu C, Appella E, Hunt DF (2006). Proteomic and bioinformatic characterization of the biogenesis and function of melanosomes. J Proteome Res.

[68] Noskova L, Stranecky V, Hartmannova H, Pristoupilova A, Baresova V, Ivanek R, Hulkova H, Jahnova H, van der Zee J, Staropoli JF, Sims KB, Tyynela J, Van Broeckhoven C, Nijssen PC, Mole SE, Elleder M, Kmoch S (2011). Mutations in DNAJC5, encoding cysteine-string protein alpha, cause autosomal-dominant adult-onset neuronal ceroid lipofuscinosis. Am J Hum Genet.

[69] Reinders Y, Schulz I, Gräf R, Sickmann A (2006). Identification of novel centrosomal proteins in *Dictyostelium discoideum* by comparative proteomic approaches. J Proteome Res.

[70] Gotthardt D, Blancheteau V, Bosserhoff A, Ruppert T, Delorenzi M, Soldati T (2006). Proteomics fingerprinting of phagosome maturation and evidence for the role of a Galpha during uptake. Mol Cell Proteomics.

[71] Cannelli N, Nardocci N, Cassandrini D, Morbin M, Aiello C, Bugiani M, Criscuolo L, Zara F, Striano P, Granata T, Bertini E, Simonati A, Santorelli FM (2007). Revelation of a novel CLN5 mutation in early juvenile neuronal ceroid lipofuscinosis. Neuropediatrics.

[72] Pineda-Trujillo N, Cornejo W, Carrizosa J, Wheeler RB, Munera S, Valencia A, Agudelo-Arango J, Cogollo A, Anderson G, Bedoya G, Mole SE, Ruíz-Linares A (2005). A CLN5 mutation causing an atypical neuronal ceroid lipofuscinosis of juvenile onset. Neurology.

[73] Xin W, Mullen TE, Kiely R, Min J, Feng X, Cao Y, O’Malley L, Shen Y, Chu-Shore C, Mole SE, Goebel HH, Sims K (2010). CLN5 mutations are frequent in juvenile and late-onset non-Finnish patients with NCL. Neurology.

[74] Lebrun AH, Storch S, Ruschendorf F, Schmiedt ML, Kyttala A, Mole SE, Kitzmuller C, Saar K, Mewasingh LD, Boda V, Kohlschutter A, Ullrich K, Braulke T, Schulz A (2009). Retention of lysosomal protein CLN5 in the endoplasmic reticulum causes neuronal ceroid lipofuscinosis in Asian sibship. Hum Mutat.

[75] Isosomppi J, Vesa J, Jalanko A, Peltonen L (2002). Lysosomal localization of the neuronal ceroid lipofuscinosis CLN5 protein. Hum Mol Genet.

[76] Moharir A, Peck SH, Budden T, Lee SY (2013). The role of N-Glycosylation in folding, trafficking, and functionality of lysosomal protein CLN5. PLoS One.

[77] Stoka V, Turk V, Turk B. Lysosomal cathepsins and their regulation in aging and neurodegeneration. Ageing Res Rev. 2016;S1568-1637(16)30067-8. 10.1016/j.arr.2016.04.010 27125852

[78] Steinfeld R, Reinhardt K, Schreiber K, Hillebrand M, Kraetzner R, Bruck W, Saftig P, Gartner J (2006). Cathepsin D deficiency is associated with a human neurodegenerative disorder. Am J Hum Genet.

[79] Vetvicka V, Fusek M (2012). Procathepsin D as a tumor marker, anti-cancer drug or screening agent. Anticancer Agents Med Chem.

[80] Didangelos A, Yin X, Mandal K, Baumert M, Jahangiri M, Mayr M (2010). Proteomics characterization of extracellular space components in the human aorta. Mol Cell Proteomics.

[81] Journet A, Chapel A, Jehan S, Adessi C, Freeze H, Klein G, Garin J (1999). Characterization of *Dictyostelium discoideum* cathepsin D. J Cell Sci.

[82] Harris E, Wang N, Wu Wl WL, Weatherford A, De Lozanne A, Cardelli J (2002). *Dictyostelium* LvsB mutants model the lysosomal defects associated with Chediak-Higashi syndrome. Mol Biol Cell.

[83] Hagedorn M, Soldati T (2007). Flotillin and RacH modulate the intracellular immunity of *Dictyostelium* to *Mycobacterium marinum* infection. Cell Microbiol.

[84] Galardi-Castilla M, Pergolizzi B, Bloomfield G, Skelton J, Ivens A, Kay RR, Bozzaro S, Sastre L (2008). SrfB, a member of the Serum Response Factor family of transcription factors, regulates starvation response and early development in *Dictyostelium*. Dev Biol.

[85] Koch S, Scifo E, Rokka A, Trippner P, Lindfors M, Korhonen R, Corthals GL, Virtanen I, Lalowski M, Tyynelä J (2013). Cathepsin D deficiency induces cytoskeletal changes and affects cell migration pathways in the brain. Neurobiol Dis.

[86] Sillo A, Matthias J, Konertz R, Bozzaro S, Eichinger L (2011). *Salmonella typhimurium* is pathogenic for *Dictyostelium* cells and subverts the starvation response. Cell Microbiol.

[87] Carrasco-Marín E, Madrazo-Toca F, de los Toyos JR, Cacho-Alonso E, Tobes R, Pareja E, Paradela A, Albar JP, Chen W, Gomez-Lopez MT, Alvarez-Dominguez C (2009). The innate immunity role of cathepsin-D is linked to Trp-491 and Trp-492 residues of listeriolysin O. Mol Microbiol.

[88] Mir H, Rajawat J, Begum R (2012). Staurosporine induced poly (ADP-ribose) polymerase independent cell death in *Dictyostelium discoideum*. Indian J Exp Biol.

[89] Rajawat J, Alex T, Mir H, Kadam A, Begum R (2014). Proteases involved during oxidative stress-induced poly(ADP-ribose) polymerase-mediated cell death in *Dictyostelium discoideum*. Microbiology.

[90] Johansson AC, Steen H, Ollinger K, Roberg K (2003). Cathepsin D mediates cytochrome c release and caspase activation in human fibroblast apoptosis induced by staurosporine. Cell Death Differ.

[91] Hah YS, Noh HS, Ha JH, Ahn JS, Hahm JR, Cho HY, Kim DR (2012). Cathepsin D inhibits oxidative stress-induced cell death via activation of autophagy in cancer cells. Cancer Lett.

[92] Kruman I, Guo Q, Mattson MP (1998). Calcium and reactive oxygen species mediate staurosporine-induced mitochondrial dysfunction and apoptosis in PC12 cells. J Neurosci Res.

[93] Zhang XD, Gillespie SK, Hersey P (2004). Staurosporine induces apoptosis of melanoma by both caspase-dependent and -independent apoptotic pathways. Mol Cancer Ther.

[94] Olie RA, Durrieu F, Cornillon S, Loughran G, Gross J, Earnshaw WC, Golstein P (1998). Apparent caspase independence of programmed cell death in *Dictyostelium*. Curr Biol.

[95] Roisin-Bouffay C, Luciani MF, Klein G, Levraud JP, Adam M, Golstein P (2004). Developmental cell death in *Dictyostelium* does not require paracaspase. J Biol Chem.

[96] Benussi A, Padovani A, Borroni B (2015). Phenotypic heterogeneity of monogenic frontotemporal dementia. Front Aging Neurosci.

[97] Petkau TL, Leavitt BR (2014). Progranulin in neurodegenerative disease. Trends Neurosci.

[98] Bublitz MJ, Morth JP, Nissen P (2011). P-type ATPases at a glance. J Cell Sci.

[99] Ramirez A, Heimbach A, Grundemann J, Stiller B, Hampshire D, Cid LP, Goebel I, Mubaidin AF, Wriekat AL, Roeper J, Al-Din A, Hillmer AM, Karsak M, Liss B, Woods CG, Behrens MI, Kubisch C (2006). Hereditary parkinsonism with dementia is caused by mutations in ATP13A2, encoding a lysosomal type 5 P-type ATPase. Nat Genet.

[100] Covy JP, Waxman EA, Giasson BI (2012). Characterization of cellular protective effects of ATP13A2/PARK9 expression and alterations resulting from pathogenic mutants. J Neurosci Res.

[101] Park JS, Blair NF, Sue CM (2015). The role of ATP13A2 in Parkinson’s disease: Clinical phenotypes and molecular mechanisms. Mov Disord.

[102] Lelong E, Marchetti A, Guého A, Lima WC, Sattler N, Molmeret M, Hagedorn M, Soldati T, Cosson P (2011). Role of magnesium and a phagosomal P-type ATPase in intracellular bacterial killing. Cell Microbiol.

[103] Cosson P, Lima WC (2014). Intracellular killing of bacteria: is *Dictyostelium* a model macrophage or an alien?. Cell Microbiol.

[104] Koller B, Schramm C, Siebert S, Triebel J, Deland E, Pfefferkorn AM, Rickerts V, Thewes S (2016). *Dictyostelium discoideum* as a novel host system to study the interaction between phagocytes and yeasts. Front Microbiol.

[105] Le Coadic M, Froquet R, Lima WC, Dias M, Marchetti A, Cosson P (2013). Phg1/TM9 proteins control intracellular killing of bacteria by determining cellular levels of the Kil1 sulfotransferase in *Dictyostelium*. PLoS One.

[106] Kaakinen R, Lindstedt KA, Sneck M, Kovanen PT, Oörni K (2007). Angiotensin II increases expression and secretion of cathepsin F in cultured human monocyte-derived macrophages: an angiotensin II type 2 receptor-mediated effect. Atherosclerosis.

[107] Pears CJ, Mahbubani HM, Williams JG (1985). Characterization of two highly diverged but developmentally co-regulated cysteine proteinase genes in *Dictyostelium discoideum*. Nucleic Acids Res.

[108] Driscoll DM, Williams JG (1987). Two divergently transcribed genes of *Dictyostelium discoideum* are cyclic AMP-inducible and coregulated during development. Mol Cell Biol.

[109] Williams JG, North MJ, Mahbubani H (1985). A developmentally regulated cysteine proteinase in *Dictyostelium discoideum*. EMBO J.

[110] Na J, Tunggal B, Eichinger L (2007). STATc is a key regulator of the transcriptional response to hyperosmotic shock. BMC Genomics.

[111] Bakthavatsalam D, Brazill D, Gomer RH, Eichinger L, Rivero F, Noegel AA (2007). A G protein-coupled receptor with a lipid kinase domain is involved in cell-density sensing. Curr Biol.

[112] Riyahi TY, Frese F, Steinert M, Omosigho NN, Glöckner G, Eichinger L, Orabi B, Williams RS, Noegel AA (2011). RpkA, a highly conserved GPCR with a lipid kinase domain, has a role in phagocytosis and anti-bacterial defense. PLoS One.

[113] Shi GP, Bryant RA, Riese R, Verhelst S, Driessen C, Li Z, Bromme D, Ploegh HL, Chapman HA (2000). Role for cathepsin F in invariant chain processing and major histocompatibility complex class II peptide loading by macrophages. J Exp Med.

[114] Staropoli JF, Karaa A, Lim ET, Kirby A, Elbalalesy N, Romansky SG, Leydiker KB, Coppel SH, Barone R, Xin W, MacDonald ME, Abdenur JE, Daly MJ, Sims KB, Cotman SL (2012). A homozygous mutation in KCTD7 links neuronal ceroid lipofuscinosis to the ubiquitin-proteasome system. Am J Hum Genet.

[115] Liu Z, Xiang Y, Sun G (2013). The KCTD family of proteins: structure, function, disease relevance. Cell Biosci.

[116] O’Day DH, Keszei A (2012). Signalling and sex in the social amoebozoans. Biol Rev Camb Philos Soc.

[117] Muramoto T, Suzuki K, Shimizu H, Kohara Y, Kohriki E, Obara S, Tanaka Y, Urushihara H (2003). Construction of a gamete-enriched gene pool and RNAi-mediated functional analysis in *Dictyostelium discoideum*. Mech Dev.

[118] Muramoto T, Takeda S, Furuya Y, Urushihara H (2005). Reverse genetic analyses of gamete-enriched genes revealed a novel regulator of the cAMP signaling pathway in *Dictyostelium discoideum*. Mech Dev.

[119] Persaud-Sawin DA, Mousallem T, Wang C, Zucker A, Kominami E, Boustany RM (2007). Neuronal ceroid lipofuscinosis: a common pathway?. Pediatr Res.

[120] Faix J, Linkner J, Nordholz B, Platt JL, Liao XH, Kimmel AR (2013). The application of the Cre-loxP system for generating multiple knock-out and knock-in targeted loci. Methods Mol Biol.

